# Supplementary material for: Prediction of malignant transformation in oral epithelial dysplasia using infrared absorbance spectra
Source: PLoS One. 2022 Mar 25;17(3):e0266043. doi: 10.1371/journal.pone.0266043 (PMC8956195; doi:10.1371/journal.pone.0266043)
Supplement: Attachment — Submitted filename: Ellis et al Response to reviewers.docx [file pone.0266043.s001.docx]

**Professor Richard Shaw MD FDS FRCS(OMFS)**

**Professor of Head & Neck Surgery, Liverpool Head & Neck Centre**

Chair: Mersey Head & Neck Oncology Research Group,

Cancer Research Centre, London Road, University of Liverpool, L3 9TA

T 0151 794 8832 E [richard.shaw@liv.ac.uk](mailto:richard.shaw@liv.ac.uk)

@livHeadandNeck

**Honorary Consultant in Maxillofacial / Head & Neck Surgery**

Liverpool University Hospitals NHS Foundation Trust,

Aintree Hospital, Longmoor Lane, Liverpool L9 7AL

Secretary: Mrs Debbie Scott

T	0151 529 5290	E	DEBBIE.SCOTT@liverpoolft.nhs.uk

4^th^ March 2021

Professor Patrick Ha

Academic Editor

PLoSONE

Dear Patrick

**RE: Revision 1 - Prediction of malignant transformation in oral epithelial dysplasia using infrared absorbance spectra. Ellis et al.**

Thank you for considering this revised manuscript for publication in PLoSONE.

We have addressed the reviewers’ and academic editor’s comments below and deposited our minimal dataset in the University of Liverpool Data Catalogue at <https://doi.org/10.17638/datacat.liverpool.ac.uk/1622>

In this work we demonstrate that the analysis of infrared microscopic data can potentially perform better than histopathology and clinical observation in the determination of the transformative capacity of oral epithelial dysplasia, a premalignant condition in the mouth. The novelty of the data presented in this manuscript is that we have used the clinically important endpoint of transformation to oral cancer rather than simply measuring the performance of the model at discriminating histopathological grade. Furthermore, we have utilized Bayesian optimization to ascertain the most effective data pre-processing procedure rather than selecting steps at random. Our data, albeit from a relatively small sample set, shows a sensitivity of 79(± 5)% and a specificity of 76(± 5)% at the level of the individual lesion, a significant improvement to the 24-40% accuracy of histopathological grade alone.

Yours sincerely,

Richard

**Response to reviewers**

We thank the reviewers for their comments and note that they are supportive of our attempt to identify a new methodology for tackling the ongoing clinical problem of identifying the transformative potential of premalignant oral lesions.

We present a listed discussion of the points raised by the reviewers and academic editor.

Reviewer 1

No comments to address. This reviewer advocates publication in the current form.

Reviewer 2

*1. From a clinical perspective, while it is helpful to understand which OED with severe histopathology undergo malignant transformation for counseling purposes, from an intervention standpoint, this is less relevant as most surgeons will excise forms of severe dysplasia. It would be of greater clinical utility to also include lesions that are mild and/or moderate dysplasia as a separate analysis, as there is more ambiguity in clinical decision making.*

The reviewer is correct, that we would usually excise severe dysplasia. However, firstly, in around 60-70% of cases this is overtreatment as this proportion of high-risk lesions don’t transform, so just because it is current standard of care does not mean that it will not be improved upon. Secondly, the stakes are high for some lesions as they are very large/multisite and often only monitored even though felt to be high risk – in these cases more certainty around prognosis would help justify invasive treatment if it was needed.

Furthermore, because of the acknowledged uncertainty in predicting transformation of moderate dysplasia in particular, we utilized lesions with severe and moderate dysplasia in our study, as noted on line 111-112 in Methods. We also noted that ‘there was no significant difference between the distribution of severe and moderate grade OED lesions in the two groups.’ (lines 119-121 in Methods).

The reviewer also correctly indicates that to inform clinical utility, a more heterogenous sample set would need to be used, for example including lower risk lesions with mild dysplasia. However, the aim of the current study was to demonstrate proof-of-principle for the novel methodology, and we would not claim that this data currently has clinical utility; we now aim to similarly analyze a much larger, more heterogenous sample set*.*

*2. The authors do acknowledge that the small sample size is a limitation of this study. However, with testing of 648 different pipelines and 17 samples, there is a concern that the authors are overfitting their data, thereby making it difficult to interpret the utility of this algorithm. It would be helpful to see the predictive accuracy of this methodology replicated with a validation cohort.*

We are in agreement with the reviewer regarding the size of the study and one of our original concerns was for overfitting when using such a small sample size. However, we wish to reiterate that the models were always trained on one dataset and then tested on a separate dataset. In order to address the possibility of a single lesion biasing the analysis, we used the ‘leave one pair out cross validation’ (LOPOCV) routine as described in Methods (lines 211-214), both for selecting the optimal pipeline and for building and testing the model. In this routine, 70 separate testing (or validation) datasets were constructed by setting aside the data from 1 non-transforming plus 1 transforming lesion in every possible combination. The training dataset for each combination was then composed of data from the remaining 6 non-transforming and 9 transforming lesions. The best data processing pipeline was defined as that which gave the largest *test mean* Matthew’s correlation coefficient across the 70 iterations of training datasets (i.e. the pipeline that performed best across all 70 training sets rather than in any one training set). The *single* pipeline identified from this optimization of processing steps was then applied to the 70 test-train sets in order to obtain specificity, sensitivity and ROC data. It should also be remembered that although there are 17 patients, each sample comprised more than 3,800 datapoints that were used in the analysis.

Thus, we acknowledge the small sample size and the need for validation in a larger sample set (lines 387-395, 419-426 & 434-440 in Discussion), but have used the mean of the 70 test-train iterations to reach some preliminary conclusions about the possible utility of the method.

The Methods section, lines 211-214, have been modified to clarify this point:

‘To assess the performance of each pipeline, 70 training sets were created by using a leave-one-pair-out cross validation (LOPOCV) method: pairs of samples (comprising 1 T and 1 NT sample in every possible combination) was set aside as the test set, while data from the remaining 6 NT and 9T samples was used for training. ‘

*3. Did all lesions undergo only incisional biopsy and then were followed? Or did some of these lesions undergo curative surgical excision?*

As stated on line 132-133 in Methods, ‘None of these NT lesions had been excised during the follow-up period.’ All patient notes were reviewed by a clinician. However, we also noted in the discussion that ‘The identification of areas predicted to have transforming capacity in one NT lesion is more difficult to explain, as none of the NT lesions were excised during the follow-up period’ (lines 365-368) and we hypothesized ‘that very small islands of putatively transforming OED could have been completely excised during the biopsy procedure, unintentionally performing a therapeutic excisional biopsy despite the intention for diagnostic incisional biopsy.’ (368-371 in Discussion).

With regard to the transforming lesions, ‘incisional biopsy material was obtained from each of 10 patients at the closest timepoint to transformation’ (Methods, lines 127-128).

*4. Please clarify how the region of interest was determined.*

We state in Methods that ‘the area of dysplasia corresponding to the histopathologically most extreme OED present was identified in each H&E-stained section and marked as the target for FTIR spectroscopy. This area was termed the region of interest (ROI).’ (lines 138-141).

Academic Editor

1. The manuscript has been checked against PLOS ONE style templates

2. The funding statement in the Acknowledgement section of the manuscript has been removed. The Funding Statement in the electronic submission is correct.

3. *Upon re-submitting your revised manuscript, please upload your study’s minimal underlying data set as either Supporting Information files or to a stable, public repository and include the relevant URLs, DOIs, or accession numbers within your revised cover letter. …. Any potentially identifying patient information must be fully anonymized. Note that it is not acceptable for the authors to be the sole named individuals responsible for ensuring data access.*

The minimal dataset has been placed in the University of Liverpool Data Catalogue, accession number: <https://doi.org/10.17638/datacat.liverpool.ac.uk/1622> (as stated above in the cover letter).

4. *While revising your submission, please upload your figure files to the Preflight Analysis and Conversion Engine (PACE) digital diagnostic tool,* [*https://pacev2.apexcovantage.com/*](https://pacev2.apexcovantage.com/)*. PACE helps ensure that figures meet PLOS requirements.*

This has been addressed. The PACE-derived figures have been uploaded with the revised manuscript.
